# Editorial: Marine microbiomes: towards standard methods and Best Practices

**DOI:** 10.3389/fmicb.2023.1219958

**Published:** 2023-06-01

**Authors:** Kelly D. Goodwin, Anaïs Lacoursière-Roussel

**Affiliations:** ^1^National Oceanic and Atmospheric Administration, NOAA Ocean Exploration, Stationed at SWFSC, La Jolla, CA, United States; ^2^St. Andrews Biological Station, Fisheries and Oceans Canada, St. Andrews, NB, Canada; ^3^Biological Sciences, University of New Brunswick, Saint John, NB, Canada

**Keywords:** microbiome, eDNA, standards and codes of practice, Best Practices, marine microbial biodiversity

The microbiome is key to understanding and sustaining the services that ocean ecosystems provide (Bolhuis et al., 2020). The marine microbiome—an ensemble of microscopic organisms that inhabit water columns, sediments, and aquatic organisms—contains members spanning in size from viruses of a few tens of nanometers to metazoans of several centimeters. Together, the microbiome forms the base of the food web, maintains animal health, and regulates most fluxes of energy and matter. Marine microbiome discovery is part of a great campaign to explore the earth's oceans, and rapid advances in high throughput sequencing are allowing a glimpse into this hidden world (Figure 1). Furthermore, these techniques have been adapted to detect DNA in the environment (eDNA) from organisms of all trophic levels.

**Figure 1:**
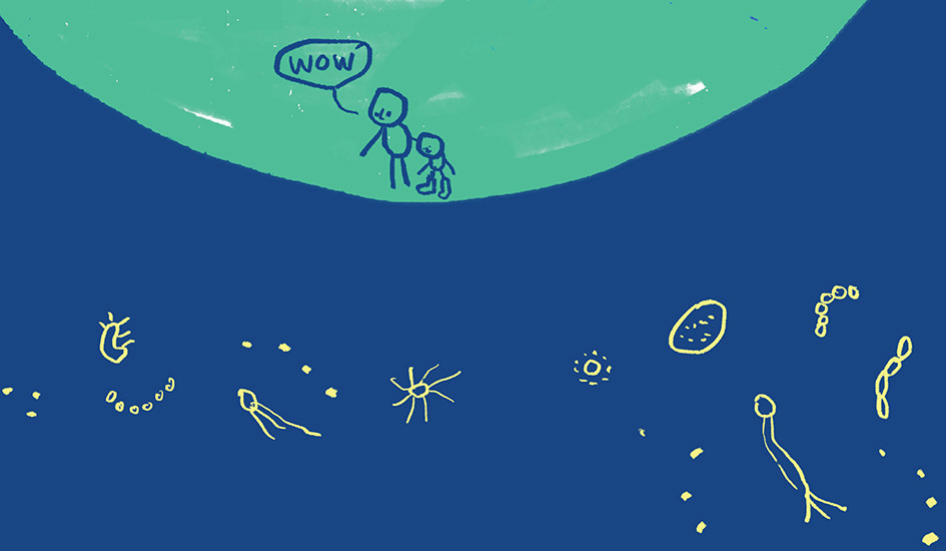
The marine microbiome is a largely unexplored treasure for society. Illustration credit: Rán Flygenring.

Biomolecular observations can provide important insights into ecosystem structure and function, development of new indicators of ecosystem health, and warnings of potential hazards to living resources and humans. Endorsements by the United Nations Ocean Decade^1^ reflect the growing demand for affordable, large-scale biological observations provided by biomolecule detection. Examples include the Ocean Biomolecular Observing Network (OBON) Program (Leinen et al., 2022), which aims to transform how we sense, harvest, protect and manage ocean life. OBON actions supporting these aims include the Observing and Promoting Atlantic Microbiomes^2^ project hosted by the Atlantic Ocean Research Alliance (AORA) Marine Microbiome Working Group^3^ (Bolhuis et al., 2020) that called for this Research Topic.

This Frontiers Research Topic was motivated by the recognition that a number of cross-cutting challenges need to be addressed to fully unlock the marine microbiome for environmental and societal benefit. Such challenges include the development and adoption of standards, common methods, Best Practices, and FAIR (Findable, Accessible, Interoperable, and Re-usable) data principles (Bolhuis et al., 2020). For some authors, the emphasis was on cyberinfrastructure to ensure that both sequence and environmental data are FAIR (Blumberg et al.). Others focused on developing a Minimum Information for an Omic Protocol (MIOP) and a public repository of protocols that can be both searched and prioritized for use (Samuel et al.). Both of these manuscripts highlighted the importance of machine readable data and products to the achievement of FAIR principles. The need to implement and sustain a global and publicly supported platform to share, discover, and compare practices and protocols was emphasized.

Papers in this Research Topic highlighted that harmonization across the full workflow—from methods through data reporting—is needed to achieve global scale biodiversity observations that can be integrated over space and time. Some manuscripts offered general overviews and “tricks of the trade” to guide microbiome sample collection and processing for coral tissues (Silva et al.) or pelagic waters for a variety of molecular targets and size fractions (Patin and Goodwin). These papers reviewed methods for multiple sections of the overall workflow with detailed guidance provided for sample collection, preservation, and processing. Other manuscripts focused on specific details, such as DNA isolation. For example, Wietz et al. described extraction of DNA from samples preserved in formalin or HgCl_2_, preservatives commonly used in sediment trap studies. Korlević et al. described a procedure to specifically isolate DNA and protein from macrophyte epiphytic communities to avoid overwhelming microbiome samples with host DNA. Gu et al. described a new analytical protocol to determine Protoporphyrin IX (PPIX) in microbial cells and provided results with coastal aquatic samples to demonstrate the potential to use PPIX as an indicator of microbial productivity. This diversity of topics underscores the large range of microbiome applications.

The growth of publicly available sequence data has increased the ability to perform meta-analysis to investigate broad scale environmental change. However, the rapid expansion of molecular techniques has created disparate protocols and workflows. A number of authors thus addressed the question of whether datasets can be combined across studies by exploring the sensitivity of taxonomic annotation to variations in sequencing methods. For example, taxonomic assignments were compared for the 16S rRNA gene V3-V4 and V4-V5 primer sets as applied to a variety of sample types collected from Arctic Ocean marine systems. In this case, V4-V5 was recommended due to superior inclusion of archaeal taxa (Fadeev et al.). In another case, a single primer set was applied to coral tissues that were processed separately (DNA extraction through library preparation) and then sequenced on different platforms (MiSeq and HiSeq). Despite past studies suggesting that MiSeq and HiSeq data could be combined to provide microbiome taxonomic analysis, the study here cautioned that significant differences in compositional assignments could arise from protocol variations (Epstein et al.). This work suggested that projects that seek to understand and overcome sources of technical variation remain needed. Multiple studies also highlighted the continued need to build out reference databases to improve annotation of sequence data.

This Research Topic fostered cross-community exchange of standards and Best Practices. It provided an opportunity for different communities working on marine microbiomes to communicate the advantages and limitations of various sampling, laboratory, and data processing methods and to open community discussion on how to move toward large scale operationalization. Although the need for harmonization was recognized, workflows must be fit for purpose; meaning that they must meet the objectives and logistical constraints of a study, application, management objective, or time series (including legacy sampling). Even with Best Practices in hand, pilot studies must be conducted to validate and optimize workflows against different sample types, geographies, or molecular targets. To support international efforts to develop guidance on Best Practices, a centralized platform could compile protocols, metadata, and data produced by specific methods. Such could include successful, unsuccessful, anecdotal, or unpublished information to provide real-world feedback. Machine Learning approaches could potentially help define optimal workflows from the growing observations. Overall, the pursuit of cross-community standards and Best Practices will foster data integration across heterogeneous methods, improve future ocean observations, and expand the trusted use of microbiome and eDNA science.

## Author contributions

All authors contributed to the writing and editing of the manuscript.

## Footnotes

^1^ https://oceandecade.org/

^2^ https://oceandecade.org/actions/ocean-biomolecular-observing-network-obon/

^3^ https://www.marinemicrobiome.org/
